# Avian paramyoxvirus-8 immunization reduces viral shedding after homologous APMV-8 challenge but fails to protect against Newcastle disease

**DOI:** 10.1186/1743-422X-11-179

**Published:** 2014-10-08

**Authors:** Christian Grund, Constanze Steglich, Eva Huthmann, Martin Beer, Thomas C Mettenleiter, Angela Römer-Oberdörfer

**Affiliations:** Institute of Diagnostic Virology, Friedrich-Loeffler-Institut, Federal Research Institute for Animal Health, Südufer 10, D-17493 Greifswald-Insel Riems, Germany; Institute of Molecular Virology and Cell Biology, Friedrich-Loeffler-Institut, Federal Research Institute for Animal Health, Südufer 10, D-17493 Greifswald-Insel Riems, Germany

**Keywords:** APMV-8, APMV, NDV, Newcastle disease, Cross-reactivity, Cross-protection

## Abstract

**Background:**

Protection against infection by Newcastle disease virus (NDV), also designated as avian paramyxovirus subtype-1 (APMV-1), is mediated by immune responses to the two surface glycoproteins, hemagglutinin-neuraminidase (HN) and fusion (F) protein. Thus, a chimeric APMV-1 based vaccine that encodes APMV-8 HN- and F-proteins and expresses the hemagglutinin of avian influenza virus (AIV) H5N1, is able to protect against HPAIV H5N1 but fails to protect against NDV [*PLoS One***8:**e72530, 2013]. However, it is unclear whether avirulent APMV-subtypes, like APMV-8 can induce subtype-specific immunity and protect from a homologous challenge.

**Findings:**

APMV-8 infections of 3- and 6-weeks-old specific pathogen free (SPF)-chickens did not induce any clinical signs but was associated with virus shedding for up to 6 days. Viral replication was only detected in oropharyngeal- and never in cloacal swabs. Upon reinfection with homologous APMV-8, viral shedding was restricted to day 2 and in contrast to naive SPF-chickens, only RNA but no infectious virus was recovered. No protection was induced against virulent NDV challenge, although morbidity and mortality was delayed in APMV-8 primed chickens. This lack of protection is in line with a lack of reactivity of APMV-8 specific sera to APMV-1 HN-protein: Neither by hemagglutin-inhibition (HI) test nor immunoblot analyses, cross-reactivity was detected, despite reactivity to internal proteins.

**Conclusions:**

Immune responses mounted during asymptomatic APMV-8 infection limit secondary infection against homologues reinfection and facilitates a delay in the onset of disease in a subtype independent manner but is unable to protect against Newcastle disease, a heterologous APMV-subtype.

**Electronic supplementary material:**

The online version of this article (doi:10.1186/1743-422X-11-179) contains supplementary material, which is available to authorized users.

## Background

Avian paramyxoviruses (APMV) replicate within the respiratory tract and intestine of their natural avian host. They belong to the genus *Avulavirus* in the family *Paramyxoviridae* within the order *Mononegavirales* [1]. Currently, 12 subtypes have been identified with APMV-1 to -9 known as ‘classical strains’ [2] and APMV-10 to 12 recently described [3–5]. The prototypic virus, APMV-1 or Newcastle Disease virus (NDV) causes a devastating disease in poultry and represents a major threat for poultry production in the world. In contrast, the other APMV-subtypes are not clinically relevant for poultry and circulate largely unnoticed in wild birds [6]. Also for APMV-1, strains of low virulence are well known. They do not induce clinical signs in immune competent birds but confer protection against ND [7–9]. The generation of recombinant NDV (rNDV) containing specific alterations in the genome decreased residual virulence [10] and were also used as vector system to express genes of other pathogens, e.g. highly pathogenic avian influenza virus (HPAIV) [11, 12]. To avoid interference by maternal NDV antibodies with vaccine vector performance, we created a chimeric virus (*ch*NDV*FHN*PMV8*H5*) by substituting the envelope glycoproteins hemagglutinin-neuraminidase (HN) and fusion protein (F) of NDV by those of APMV-8 and expressing H5 of HPAIV [13]. APMV-8 was chosen as donor of HN and F because of its apathogenicity for poultry [14], description of only weak cross-reactivity between APMV-1 and APMV-8 [15, 16] and low prevalence [17–19]. Vaccination with this chimeric vector HPAIV-H5-vaccine resulted in efficient protection against HPAIV H5N1 infection in chickens with NDV specific maternal antibodies (MDA) [1]. However, evasion from maternal NDV antibodies was accompanied by a lack of protection against ND. This observation corresponds to investigations by Nayak et al. [20] describing absence of protection against NDV challenge after APMV-8 infection. The serological data of the vaccination experiments with *ch*NDV*FHN*PMV8*H5* as well as the APMV-8 infection suggested that APMV-8 glycoproteins were immunogenic in the host. However, it remained unclear whether an APMV-8 immune response was sufficient to induce protection against homologous APMV-8 challenge. Here we describe a set of animal experiments demonstrating, that APMV-8 infection does not prevent subsequent reinfection with homologous APMV-8 but limits viral shedding.

## Materials and methods

### Animal experiments

Chickens from SPF-eggs (LAH, Cuxhaven), hatched at the FLI were infected oculo-nasally at 3 weeks of age with 0.1 ml of APMV-8/goose/Delaware/1053/76 containing 10^6^ TCID_50_ (n = 18). Two days post infection (dpi) two naive chickens were introduced as sentinel birds. On each of day 2, 4, 6 and 21 dpi two inoculated chickens were sacrificed and indicated internal organs were tested for APMV-8 by RT-qPCR (primer probe sequence see Additional file 1: Table S1). Three weeks after initial infection, groups were divided, one being challenged oculo-nasally with NDV/Herts33/56 (10^6^ EID_50_/animal) and the other re-infected oculo-nasally with APMV-8/goose/Delaware/1053/76 (10^6^ TCID_50_/animal). In addition, each of the two viruses at the same dose and route was administered to 6 naive chickens from the same hatch. Animals were scored daily according their clinical condition (0 = healthy; 1 = sick; 2 = dead) and clinical index was calculated analogous to determining intracerebral pathogenicity index (ICPI). At indicated time intervals post infection either oropharyngeal and cloacal swabs (primary infection) or combined oropharyngeal and cloacal swabs were taken for virus detection. Heparinised blood samples were obtained from all animals before vaccination, before challenge- or reinfection as well as from all surviving birds at the end of the observation period, and were tested for NDV- and APMV-8-specific antibodies using the hemagglutination inhibition (HI) assay [21]. All animal experiments were carried out in BSL3 experimental animal facilities and had been approved by the animal welfare committee (LALLF M-V/TSD/7221.3-1.1-053/10). See Additional file for specification of viruses (Additional file 2: Table S2) and APMV-subtype specific sera with their degree of cross-reactivity (Additional file 3: Table S3).

## Results and discussion

### APMV-8 infection

**Figure 1:**
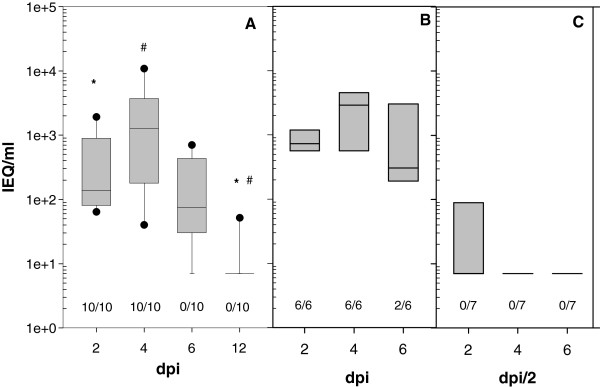
**Oropharyngeal APMV-8 shedding.** Shedding of APMV-8 was tested at indicated days after infection of 3-weeks- **(A)** and 6-weeks- **(B)** old naive SPF-chickens or after reinfection **(C)** 3 weeks after priming. Boxplots represent results of APMV-8 specific RT-qPCR with infectivity equivalents (IEQ) derived from a standard curve prepared for each PCR-run. Testing for viral RNA was done by nucleocapsid protein (NP) specific RT-qPCR following a published protocol [11] with APMV-8 specific primer and probes. Numbers indicate the number of animals per group that tested positive for infectious virus on LMH cells as described previously [22]. Differences between time-points and groups were statistically tested by appropriate Wilcoxon-tests for paired and unpaired data respectively and labeled with specific symbols. Bonferroni correction was applied in case of multiple testing. The global significance level was 0.05. All calculations were performed using R software [23], version 2.13.0(2011-04-13).

Primary APMV-8 infection was conducted in 3-weeks-old SPF chickens by installation of 10^6^ TCID_50_ of APMV-8/goose/Delaware/1053/76 into the eye and nasal cavity. Virus shedding was detected in oropharyngeal swabs of all ten investigated animals on days 2 and 4 pi, positive by RT-qPCR (Figure 1) as well as virus isolation. Later, the amount of viral RNA declined and the number of chickens shedding virus decreased to 8 and 1 on day 6 and 12, respectively. In these later swab samples, virus isolation was not successful. Virus was never detected in cloacal swabs, neither by RT-qPCR nor by virus isolation. These results of confined virus replication paralleled the observation that virus RNA was not detected in lung, liver, kidney or pancreas by RT-qPCR testing two animals sacrificed on day 2, 4, 6 and 21 pi each. Viral RNA was detected in the proventriculus of one chicken on day 2 and 4 dpi (300 and 180 genome equivalents (GEQ)/ml respectively) and in the duodenum on day 4 pi (160 GEQ/ml). Detection of a minute amounts RNA in the upper digestive tract but never in feaces points to degraded viral products rather than virus replication within these organs. These data are in agreement with observations by Kim et al. [14] detecting APMV-8 in trachea but not lung, spleen or brain of three birds sampled at day 4 after infection. Like in the former experiments, all APMV-8 infected chickens remained healthy over the entire observation period and developed like uninfected hatch mates. However, virus replication was sufficient to transmit APMV-8 to both sentinel chickens: Testing serum 21 dpi, both sentinels had seroconverted.

**Figure 2:**
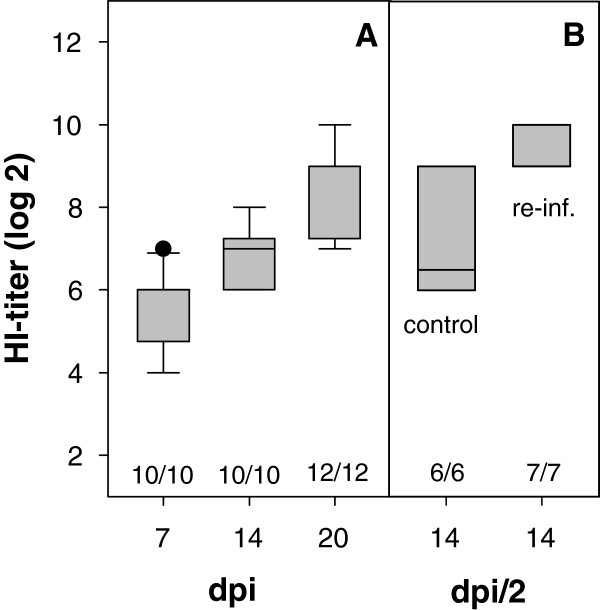
**Antibody response after APMV-8 infection.** Serum samples were taken at indicated time points after primary infection of 3-weeks- **(A)** and 6-weeks- (**B**, control) old naive SPF-chickens or after secondary APMV-8-infection (**B**, re-inf.), 3 weeks after priming. Boxplots represent HI titers, numbers indicate the number of seropositive animals per group (HI-titer >3).

**Figure 3:**
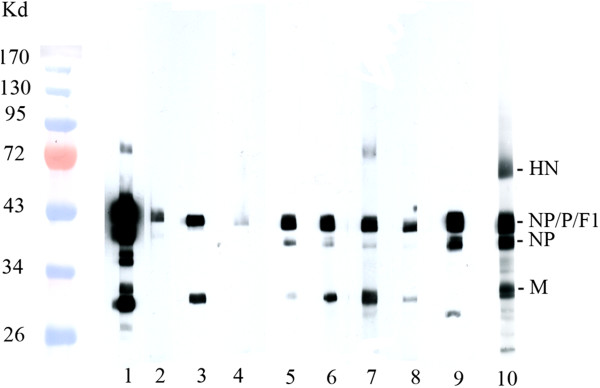
**Cross-reactivity of subtype specific immune sera with APMV-1 proteins.** Gradient purified APMV-1 strain Clone 30 were lysed, and proteins were separated by SDS-PAGE, transferred on nitrocellulose and probed by IgY-specific POD conjugated sera as described previously [24]. Identity of proteins is indicated on the right, molecular weights of marker proteins (PEQLAB™ prestained protein marker IV) are indicated on the left. Lane 1–10 show results of hyperimmune sera raised against APMV-1 (1), APMV-2 (2), APMV-3 /P (3), APMV-3/T (4), APMV-4 (5), APMV-6 (6), APMV-7 (7), APMV-8 (8), APMV-9 (9) and PPMV-1 (10), respectively. Sera from naive chickens did not result in any band (data not shown).

All APMV-8-inoculated animals seroconverted within the first week after infection and antibody titers rose to up to 8 (arithmetic mean Ø 6.9 ± 0.7) and 10 (Ø 8.6 ± 1.0) log2 two and three weeks post infection, respectively (Figure 2). Cross-reactivity of APMV-8 specific immune sera to APMV-1 HN was neither observed by HI (data not shown) nor immunoblot analyses (Figure 3). However, immunoblot analyses revealed cross-reactivity predominantly directed against internal virion proteins (Figure 3; identification of proteins see Additional file 4: Figure S1). Beside reactivity to the matrix (M) protein, a protein band of about 50–55 kD was reproducibly detected. Due to only minor differences in molecular weight amongst NP, P and the F1 subunit of the F protein, further differentiation of antibody specificity was not possible.

Three weeks after the initial experiment, five APMV-8 infected chickens and one sentinel animal, now six weeks old, were reinfected with 10^6^ TCID_50_ of homologous APMV-8 strain. In addition, a group of six chickens from the same hatch but kept separately, were APMV-8 infected. As anticipated, birds inoculated with APMV-8 did not develop clinical signs but virus shedding was detected in naive 6-weeks-old chickens from day 2 pi by RT-qPCR as well as by virus isolation comparable to shedding observed before in the 3-weeks-old animals. In contrast, in animals with previous exposure to APMV-8, virus was only detected in 3 out of 6 (47, 89 and 12087 infectivity equivalents/ml) inoculated chickens on day 2 pi and only by RT-qPCR (Figure 3). The chickens that served as sentinel for the first infection remained virus negative in all tested samples. After APMV-8 reinfection, a slight increase in antibody titer was observed, supporting the notion of limited virus-replication. It is interesting to note, that age of the animal has apparently little effect on virus replication, since virus shedding (Figure 3, A and B) and antibody response (Figure 1, A and B control) in 3- and 6-weeks-old chickens were comparable.

### Heterologous NDV-challenge

**Figure 4:**
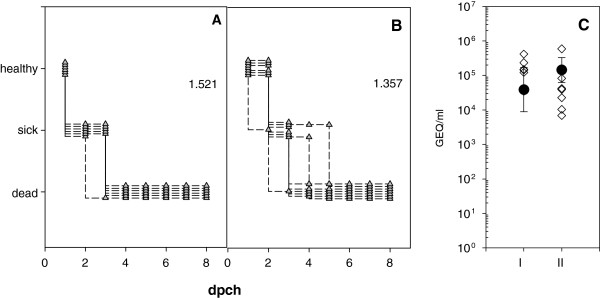
**Course of NDV challenged chickens.** Six-weeks-old naive SPF-chickens **(A)** or SPF-chickens infected/vaccinated with APMV-8 at the age of 3 weeks **(B)** were challenged with virulent NDV Herts 33/56 and monitored for clinical signs. Numbers indicate clinical index. Virus shedding was tested by NDV-specific RT-qPCR 2 days pch **(C)** for SPF-chickens (I) and APMV-8 vaccinated chickens (II). Given are genome equivalents (GEQ) derived from a standard curve prepared for each PCR-run. Differences between groups were statistically tested by appropriate Wilcoxon-tests for paired data. The global significance level was 0.05. All calculations were performed using R software [23], version 2.13.0(2011-04-13).

APMV-8 preinfected (n = 6) and naive chickens (n = 6) were challenged with NDV in parallel. All chickens suffered severe clinical disease and swab samples tested positive for virus on day 2 pch by APMV-1 specific RT-qPCR [10] as well as by virus isolation (Figure 4). All naive SPF-chickens became sick on day 2 and were dead by day 3. In animals that had previously been infected by APMV-8, the onset of disease was significantly delayed (p = 0.01515) with only one sick animal on day 2 pch and death of all chickens by day 5. Thus, clinical index of the group with APMV-8 preinfection was lower (1.36) compared to naive chickens (1.52) after NDV-challenge (Figure 4). These data support the conclusion that cross-reactivity between internal proteins has little effect on the clinical outcome after a heterologous NDV challenge, but is able to delay the clincal course. This is in agreement with earlier experiments of Nayak and colleagues [20]: They observed mortality up to day 11 in APMV-8 preinfected chickens, compared to 9 days in non-immunized control chickens, and one out of five chickens survived challenge with 200 chicken lethal dose _50_ (CLD_50_) of virulent NDV strain Texas-GB. It is interesting to note, that also after vaccination with chimeric APMV-1/APMV-8 vector vaccine (*ch*NDV*FHN*PMV8*H5*) a comparable delay of 2 days in the clinical course of ND was observed applying the same NDV challenge model [13]. Apparently, this effect of retarded clinical onset of disease is independent of the subtype. Whether this effect is associated to cross-reactivity to internal proteins needs further elucidation.

## Conclusions

Active local replication of APMV-8 induced an immune response efficacious to limit APMV-8 reinfection but unable to protect against heterologous APMV-1 subtype.

## Electronic supplementary material

Additional file 1: Table S1: Primers and probe for APMV-8 NP-specific RT-qPCR. (DOC 26 KB)

Additional file 2: Table S2: Specification and source of viruses. 1 APMV-8 strain at the FLI was obtained in 1992 and working stocks for sequencing and animal experiments were derived form the 5th and 6th passage in SPF-chicken eggs. Genome was sequenced by Müller et al. (Acc. Nr. FJ 619036). Compared to published sequences by Paldurai et al. [25] (Acc. Nr. FJ 515863) 512 Single Nucleotide Polymorphism (SNP) are evident compared to 21 SNP to pintail/Wakuya/20/78; FJ215864, [25]. (DOC 34 KB)

Additional file 3: Table S3: Cross-reactivity of APMV-subtypes tested by hemagglutination-inhibition test. Sera were produced by immunizing six-week-old SPF chickens with beta propriolactone (0.05% v/v) inactivated virus containing allantoic fluid emulsified with Freudschen adjuvant (Sigma). Sera were taken two weeks after the last of three immunizations and stored at -20°C. Results are given as log2 titer. (DOC 28 KB)

Additional file 4: Figure S1: Identification of specific APMV-1 proteins. Gradient purified APMV-1 strain Clone 30 were lysed, and proteins were separated by SDS-PAGE, transferred on nitrocellulose and probed by IgY-specific POD-conjugated sera as described previously [24]. Identity of proteins is indicated on the right, molecular weights of marker proteins (PEQLAB™ prestained protein marker IV) are indicated on the left. Lanes show results of chicken serum raised against PPMV-1 (1), monoclonal antibodies directed against NP- (2), P- (3); HN-protein( 4) and monospecific rabbit serum raised against F-protein (5). (PPT 145 KB)
