# Supplementary material for: Avian paramyoxvirus-8 immunization reduces viral shedding after homologous APMV-8 challenge but fails to protect against Newcastle disease
Source: Virol J. 2014 Oct 8;11:179. doi: 10.1186/1743-422X-11-179 (PMC4203933; doi:10.1186/1743-422X-11-179)
Supplement: Supplementary file 1 — Additional file 1: Table S1: Primers and probe for APMV-8 NP-specific RT-qPCR. (DOC 26 KB) [file 12985_2014_2506_MOESM1_ESM.doc]

Additional file 1: Table S1: Primers and probe for APMV-8 NP-specific RT-qPCR

| APMV-8np231F | 5’-GAG AGA TGG AGC CTT GCT TG-3’ |
| --- | --- |
| APMV-8np313R | 5’GAA TGT AGA CTC AGC AGT GAC A-3’ |
| probe: | 5’-[FAM]-TGA TTC ATC AAC CAC ACC AAT GAA GCA -[BHQ1] |
