# Supplementary material for: Avian paramyoxvirus-8 immunization reduces viral shedding after homologous APMV-8 challenge but fails to protect against Newcastle disease
Source: Virol J. 2014 Oct 8;11:179. doi: 10.1186/1743-422X-11-179 (PMC4203933; doi:10.1186/1743-422X-11-179)
Supplement: Supplementary file 2 — Additional file 2: Table S2: Specification and source of viruses. 1 APMV-8 strain at the FLI was obtained in 1992 and working stocks for sequencing and animal experiments were derived form the 5th and 6th passage in SPF-chicken eggs. Genome was sequenced by Müller et al. (Acc. Nr. FJ 619036). Compared to published sequences by Paldurai et al. [25] (Acc. Nr. FJ 515863) 512 Single Nucleotide Polymorphism (SNP) are evident compared to 21 SNP to pintail/Wakuya/20/78; FJ215864, [25]. (DOC 34 KB) [file 12985_2014_2506_MOESM2_ESM.doc]

Additional file 2: Table S2: Specification and source of viruses

| Virus | source |
| --- | --- |
| APMV-1/Clone 30 | MSD Animal Health |
| PPMV-1/DE/R75/98 | FLI |
| NDV/Herts33/56 | MSD Animal Health |
| APMV-2/chicken/California/Yucaipa/56 | AHVLAH, Weybridge |
| APMV-3/ turkey/England/1087/82 (APMV-3/T) | AHVLAH, Weybridge |
| APMV-3/parakeet/Netherland/449/75 (APMV-3/P) | AHVLAH, Weybridge |
| APMV-4/duck/Hongkong/D3/75 | AHVLAH, Weybridge |
| APMV-6 duck/HK/199/77 | AHVLAH, Weybridge |
| APMV-7/dove/Tu/4/75 | AHVLAH, Weybridge |
| APMV-8/ Goose/Delaware/1053/76 (Acc. no. FJ619036)1 ^1^ | Prof. Kaleta, JLU Gießen |
| APMV-9/ dom. Duck/NewYork 22/78 | AHVLAH, Weybridge |

1 APMV-8 strain at the FLI was obtained in 1992 and working stocks for sequencing and animal experiments were derived form the 5^th^ and 6^th^ passage in SPF-chicken eggs. Genome was sequenced by Müller et al. (Acc. Nr. FJ 619036). Compared to published sequences by Paldurai et al. (25) (Acc. Nr. FJ 515863) 512 Single Nucleotide Polymorphism (SNP) are evident compared to 21 SNP to pintail/Wakuya/20/78; FJ215864,(25).
