# Supplementary material for: Avian paramyoxvirus-8 immunization reduces viral shedding after homologous APMV-8 challenge but fails to protect against Newcastle disease
Source: Virol J. 2014 Oct 8;11:179. doi: 10.1186/1743-422X-11-179 (PMC4203933; doi:10.1186/1743-422X-11-179)
Supplement: Supplementary file 3 — Additional file 3: Table S3: Cross-reactivity of APMV-subtypes tested by hemagglutination-inhibition test. Sera were produced by immunizing six-week-old SPF chickens with beta propriolactone (0.05% v/v) inactivated virus containing allantoic fluid emulsified with Freudschen adjuvant (Sigma). Sera were taken two weeks after the last of three immunizations and stored at -20°C. Results are given as log2 titer. (DOC 28 KB) [file 12985_2014_2506_MOESM3_ESM.doc]

Additional file 3: Table S3: Cross-reactivity of APMV-subtypes tested by hemagglutination-inhibition test

Sera were produced by immunizing six-week-old SPF chickens with beta propriolactone (0.05 % v/v) inactivated virus containing allantoic fluid emulsified with Freudschen adjuvant (Sigma). Sera were taken two weeks after the last of three immunizations and stored at -20 °C. Results are given as log2 titer.
