# Supplementary figures and images for: Avian paramyoxvirus-8 immunization reduces viral shedding after homologous APMV-8 challenge but fails to protect against Newcastle disease
Source: Virol J. 2014 Oct 8;11:179. doi: 10.1186/1743-422X-11-179 (PMC4203933; doi:10.1186/1743-422X-11-179)

## Slide 1
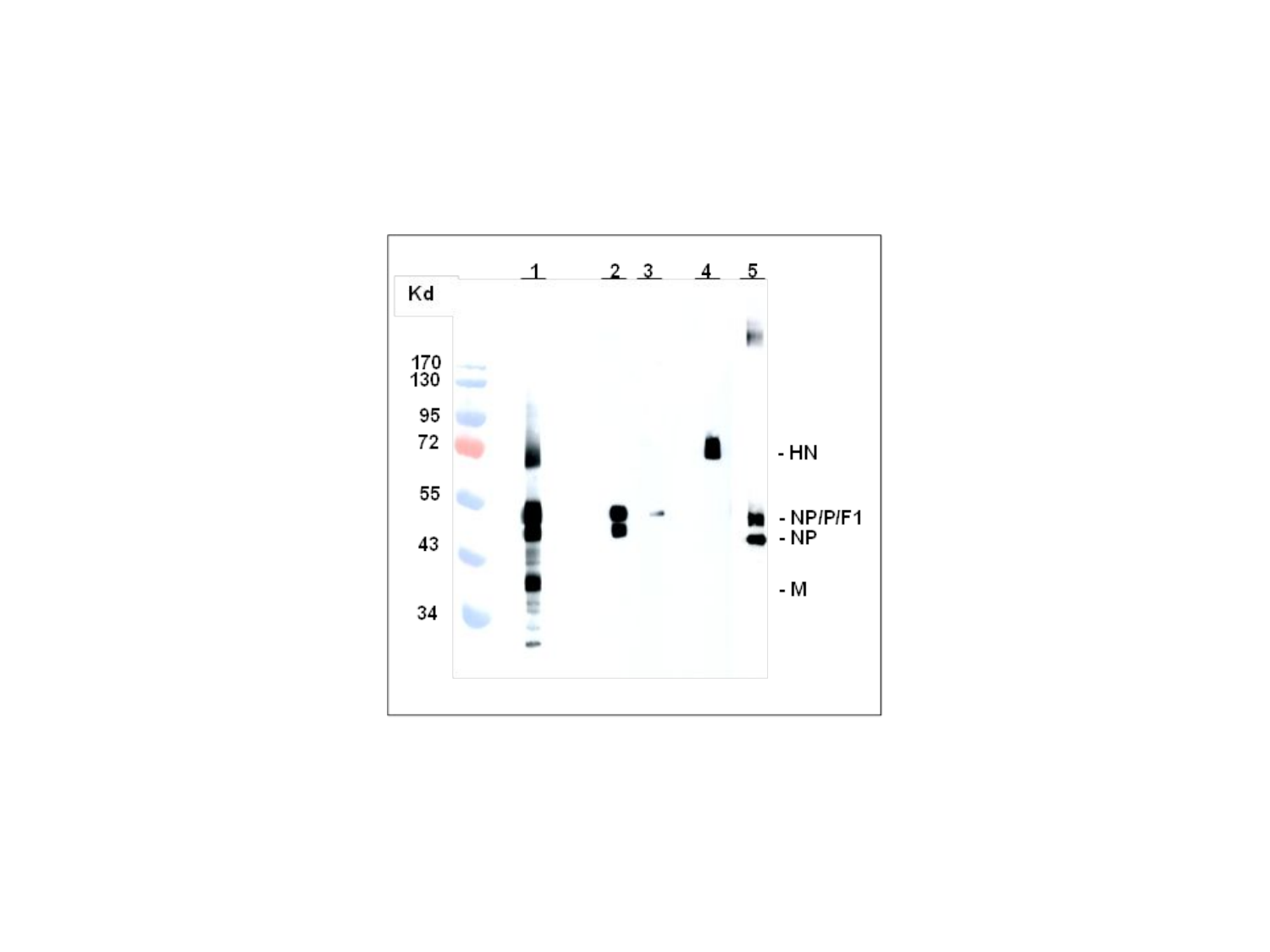

Supplement: Supplementary file 4 — Additional file 4: Figure S1: Identification of specific APMV-1 proteins. Gradient purified APMV-1 strain Clone 30 were lysed, and proteins were separated by SDS-PAGE, transferred on nitrocellulose and probed by IgY-specific POD-conjugated sera as described previously [24]. Identity of proteins is indicated on the right, molecular weights of marker proteins (PEQLAB™ prestained protein marker IV) are indicated on the left. Lanes show results of chicken serum raised against PPMV-1 (1), monoclonal antibodies directed against NP- (2), P- (3); HN-protein( 4) and monospecific rabbit serum raised against F-protein (5). (PPT 145 KB) [file 12985_2014_2506_MOESM4_ESM.ppt]
